# Case Report: Rehabilitation After Platelet-Rich Growth Factors’ Intra-Articular Injections for Knee Osteoarthritis: Two Case Reports of a Home-Based Protocol

**DOI:** 10.3389/fphar.2021.718060

**Published:** 2021-08-23

**Authors:** Francesco Negrini, Francesco De Lucia, Stefano Negrini, Davide Tornese, Francesca Facchini, Michele Vecchio, Laura de Girolamo

**Affiliations:** ^1^ IRCCS Istituto Ortopedico Galeazzi, Milan, Italy; ^2^ Department of Biomedical and Biotechnological Sciences, University of Catania, Catania, Italy; ^3^ Department of Biomedical, Surgical, and Dental Sciences, University La Statale, Milan, Italy; ^4^ Servizio di Fisioterapia, Synlab CAM, Monza, Italy; ^5^ Rehabilitation Unit, A.O.U. Policlinico S. Marco, Catania, Italy

**Keywords:** knee OA, exercise, physiotherapy, functionality, platelet-rich plasma

## Abstract

Knee osteoarthritis (KOA) is a chronic progressive disease that can cause pain, functional impairment, and ultimately disability. A novel and promising therapeutic approach to KOA is the so-called regenerative medicine, a set of procedures designed to harness tissue regenerative capacity and optimize functional recovery. Increasing evidence points out that platelet-rich plasma (PRP) intra-articular injections can decrease pain and improve functional abilities in KOA patients. In the present case reports, we analyze two patients who were treated with PRP injections coupled with a posttreatment home-based rehabilitation program. The two patients were selected to represent two different populations: patient 1 was an 85-year-old with severe impairment of functional abilities, while patient 2 was a younger (59 years old) and more active patient. The protocol consisted in a series of exercise to be performed at home, during the five days following PRP injection for two consecutive weeks (10 days in total). The exercises were designed to reduce the inflammation after the injection, enhance the proprioceptive control of the treated lower limb, and strengthen hip and knee flexors and extensors, mainly by isometric work. Results were evaluated at two time points: before and 2 months after the first PRP injection. The outcomes considered were as follows: visual analog scale for pain, EuroQol 5 dimensions questionnaire, Tegner Activity Scale for functioning, and Knee Injury and Osteoarthritis Outcome Score (KOOS). Both patients did not report any side effects from the treatment. Improvement in patient 1 was drastic at the two months follow-up as far as pain and functional abilities are concerned. Patient 2’s improvement was less evident, probably due to the higher starting point in both pain and functionality. Overall, the developed program seemed safe and was tolerated by the patients analyzed in the study, who performed it with good compliance.

## Introduction

Osteoarthritis (OA) is a chronic progressive disease that mainly affects the articular cartilage, causing pain, stiffness, and limitation of articular range of motion (ROM) (Haq et al., 2003). It consists of degeneration of the cartilage, which can also extend to the other tissues forming the joint, including synovial membrane and subchondral bone (Man and Mologhianu, 2014). While OA can affect every human joint, the knee is one of the most involved (knee osteoarthritis, KOA). Alone, it accounts for 83% of the total osteoarthritis burden (Vos et al., 2012). OA is significant in terms of epidemiologic, clinical, and socioeconomic features; it involves about 250 million individuals all over the world (Hunter et al., 2011), and about 14 million people in the United States suffer from symptomatic KOA (Deshpande et al., 2016), with an increasing trend in the last 25 years (Spitaels et al., 2020). KOA especially involves older adults; it occurs in 13% of people after age 60 years, with a high risk of mobility restriction (Hunter et al., 2011).

Multiple therapeutic approaches are currently available for KOA, including pharmacological treatments, rehabilitation (Kolasinski et al., 2020), and surgical solutions such as unicompartmental or total knee replacement (Kennedy et al., 2020). A widely used approach includes intra-articular injections, particularly with hyaluronic acid (HA), because of its relative security profile and effectiveness (Xing et al., 2016). An emerging therapeutic option is the so-called regenerative medicine, that is, a set of procedures designed to harness tissue regenerative capacity and optimize functional recovery after injury or during diseases. Regenerative medicine procedures are applied in several medical and surgical branches, like dentistry, dermatology, spine and orthopedic surgery, and rehabilitation medicine. The goal of regenerative medicine is to restore tissue homeostasis mainly by regulating anti-inflammatory processes that are responsible for most of the patients’ symptoms (Ambrosio and Rando, 2018).

In particular, blood-derived products are among the most used therapeutic options in regenerative medicine because of their efficacy together with ease of preparation and use. They consist of fluid phase plasma, with platelets, white cells, and red cells suspended in different concentrations, depending on the specific preparation protocol and indication of use (DeLong et al., 2012). Platelet-rich plasma (PRP) is the most common term used to indicate a wide category of blood-derived products containing hyperphysiological concentration of growth factors, cytokines, and bioactive factors stored in platelet α-granules (Nguyen et al., 2011; Flaumenhaft and Koseoglu, 2017).

The rationale of PRP use in patients with KOA is the high and easily accessible content of these critical growth factors and other signaling molecules, which are involved in both healing processes and immune- and inflammatory modulation, therefore potentially leading to the restoration of joint tissue homeostasis (Cole et al., 2010; Woodell-May et al., 2021). Nevertheless, the term PRP indicates a wide diversity of products, differing in preparation, composition, and application protocols. The main differences that also affect the classification of blood-derived products are platelet concentration (range 1.5–9 times above baseline) and leukocyte concentration, calling the products with low white blood cell (WBC) concentrations leukocyte-poor platelet-rich plasma (LP-PRP) and those with higher WBC concentrations as leukocyte-rich platelet-rich plasma (LR-PRP). Among them, plasma rich in growth factors (PRGF) is an autologous blood-derived product with a standardized composition and dosage. It is characterized by moderated platelet concentration (2–2.5 times compared with peripheral blood) and no WBCs. PRGF must be activated before being injected with calcium chloride to release all the growth factors.

An important classification distinguishes between leukocyte-rich PRP (LR-PRP) and leukocyte-poor PRP (LP-PRP) preparations, depending on a higher or lower concentration of leukocytes (neutrophils particularly) from the baseline (Le et al., 2018). LR-PRP could be able to increase inflammatory response: Dragoo et al. observed a clinical inflammatory pattern (peak in 5^th^ day, resolution after 2 weeks) and an increased release of MMP-3, MMP-13, IL-1b, IL-6, and TNF-α in rabbits treated with LR-PRP injections in patellar tendon (Dragoo et al., 2012). On the other hand, Yan et al. described a lower inflammatory response in terms of clinical symptoms and released inflammatory cytokines in LP-PRP intra-tendon injections in rabbit, associated to a relative better regenerative process (Yan et al., 2017). Although clinical practice is oriented in using poor formulations of leukocytes, more studies in humans are needed for a definitive choice (Lana et al., 2019).

There is increasing evidence on the efficacy of intra-articular injections of blood-derived products in patients with KOA in terms of pain reduction and improvement of function and activities of daily living (Shen et al., 2017; Hohmann et al., 2020). Rehabilitation seems to be among the best option for KOA patients. Fransen et al. described the effects of individually supervised or class exercise programs of muscle strengthening, functional training, and aerobic fitness. They found reduced knee pain and a relative improvement in physical function up to 6 months after the end of treatment (Fransen et al., 2015). Hurley et al. highlighted the positive role of land- or water-based exercise programs on pain (an absolute reduction of 6%), function (reduction of Western Ontario and McMaster Universities Osteoarthritis—WOMAC Index, from 49.9 to 44.3%), and health-related quality of life (improvement of 36-item Short Form—SF-36, by 7.9%) (Hurley et al., 2018). An interesting perspective in KOA treatment is the combination of HA intra-articular injections and rehabilitation. Block and Miller proposed an eight-week exercise program of low-impact aerobics and muscle flexibility exercises, joint mobilization, and physical therapy modalities after HA injection for symptomatic KOA patients. The authors observed increased WOMAC scores in the pain, functional, and stiffness domains in 79, 75, and 76% of patients, respectively (Block and Miller, 2013).

Currently, we are not aware of validated guidelines or trials on the association of a rehabilitation exercise-based program with PRP intra-articular injection for pain, function, and quality of life on human patients with KOA. Therefore, these case reports aim to check the feasibility and safety of a rehabilitation program following PRP intra-articular injection.

## Suggested Posttreatment and Rehabilitation Program

During the first 48 h after PRP (PRGF) injection, activities of daily living were allowed. In case of pain, 1,000 mg acetaminophen were prescribed for max three times a day, and the local application of ice for 20 min, three times a day. Instructions were given both orally and in written on a pamphlet by the clinician performing the injection (Appendix n.1).

The rehabilitation program was designed to work in synergy with the injection. It aimed to reduce the inflammation after the injection, enhance the proprioceptive control of the treated lower limb, and strengthen hip and knee flexors and extensors, mainly by isometric work. The program included three exercises, which are as follows:

− Isometric co-contractions of quadriceps and hamstrings in the supine position, maintaining knee extended: 5 repetitions lasting 10 s for three series, twice a day.
− Straight leg raises (SLR) in the supine position: 10 repetitions for three series, twice a day.
− “Alphabet,” in the supine position, maintaining hip flexed at about 45° and knee extended. In this exercise, the patient has to "draw" with his foot some letters of the alphabet, maintaining the lower limb's described position: ten letters for three series, twice a day.

After an accurate explanation, both patients performed the program during the 5 days following PRP injection for two consecutive weeks (10 days in total). We recommended not practicing sports for the entire duration of the rehabilitation program.

## Case Reports

We tested the program on two consecutive female patients recruited at RE.GA.IN (Regenerative Galeazzi Institute) Centre of IRCCS Galeazzi, Milan, Italy, between January and March 2020. The following case report adheres to the CARE (CAse REporting) structure and reporting guidelines (Gagnier et al., 2013).

### Case 1

A woman, 85 years old, with a BMI of 28.1, presented a diagnosis of left KOA, with pain onset around six months before. She had no knee traumas in the last year. She was a nonsmoker with hypertension and hypercholesterolemia, in treatment with NSAIDs, pain killers (including acetaminophen and opioid drugs), antithrombotics/antiplatelets, anticholesteremic agents, proton-pump inhibitors, and vitamin D plus calcium supplements. She already underwent a complete KOA rehabilitation program, intra-articular injections of corticosteroids and hyaluronic acid with no results.

The PRGF-Endoret® was prepared using the method described by Sanchez et al. (Sánchez et al., 2012) and administered twice intra-articularly one week apart accordingly to our personal protocol. Before each injection, the patients underwent a venous blood withdrawal of 18 ml, which was put into two extraction tubes containing the 3.8% sodium citrate anticoagulant. The blood was centrifuged at 2000 rpm (580 g) for 8 min in a BTI Biotechnology Institute system centrifuge at room temperature. After the centrifugation, the 2 ml of PRGF between the red series and the "buffy coat," the layer rich in leukocytes, was extracted by mechanical pipetting of each tube. The 2 ml of PRGF were injected into a single tube (4 ml in total). Before a knee intra-articular injection, 400 μL of calcium chloride were added for activation. Every step was conducted respecting strictly sterile conditions.

The outcome measures were as follows: visual analog scale (VAS) for pain (0–10, with 0 no pain and 10 maximum pain perceivable) (Wewers and Lowe, 1990); EuroQol 5 dimensions (EQ-5D) questionnaire, a standardized instrument that studies the quality of life (QoL) (van Reenen et al., 2020) of patients rating 5 dimensions (mobility, self-care, usual activities, pain, and anxiety/depression) with 3 possible levels of problems (1: none, 2: mild to moderate, and 3: severe); the Tegner Activity Scale for functioning (Tegner and Lysholm, 1985; Collins et al., 2011), a scale that ranges from 0, that means sick leave or disability, to 10, participation in national or international competitive sport; and Knee Injury and Osteoarthritis Outcome Score (KOOS) for evaluating function and ability to perform activities of daily living (ADL) (Roos et al., 1998; Collins et al., 2011), a test with 5 subscales (pain, symptoms, activities of daily living, function in sports and play, and knee-related quality of life) scored from 0 to 100, where 0 means bigger issues and 100 no issues. Starting from the raw values of EQ-5D, we calculated the utility value needed to estimate quality-adjusted life-years (QALYs), using the country-specific correction proposed by Scalone et al. (Scalone et al., 2013). The utility value can range from 0.00 (death) to 1.00 (perfect health). We evaluated all the outcomes at two different time points: before (T0) and two months after the first PRP injection (T1).

The pain-related score showed improvement, in terms of VAS, EQ VAS, the pain-related domain of EQ-5D, and the KOOS pain domain (Table 1). The Tegner Activity score 1) did not change after the treatment time, whereas KOOS passed from 6.6 to 60.1 points, with bigger changes in the ADL domain (from 5.88 to 60.29) and sport domain (from 0 to 80). The utility values calculated from EQ-5D scores were 0.55 at T0 and 0.77 at T1 (Table 2).

**TABLE 1:** Results of VAS for pain, EQ-5D, Tegner Scale at T0 (before injection) and T1 (two months after the first PRP injection).

	PRP type	VAS T0	VAS T1	EQ-5D T0	EQ-5D T1	EQ VAS T0	EQ VAS T1	Tegner T0	Tegner T1
**Patient 1**	PRGF-Endoret	10	8	22,231	22,221	20	70	1	1
**Patient 2**	PRGF-Endoret	4.2	4.2	11,122	11,122	53	61	4	2

**TABLE 2:** Results of the KOOS score and its domains at T0 and T1.

	KOOS Activity Scale					
	TOT	Pain	Symptoms	Function	Sports	Q.O.L
	T0	T0	T0	T0	T0	T0
**Patient 1**	6.55	2.77	17.86	5.88	0	6.25
**Patient 2**	79.76	86.11	75	92.65	60	43.75
	**T1**	**T1**	**T1**	**T1**	**T1**	**T1**
**Patient 1**	60.12	55.55	75	60.29	80	18.75
**Patient 2**	80.95	88.88	64.28	97.06	70	37.5

### Case 2

A woman, 59 years old, with a BMI of 20.3, presented a diagnosed with right KOA, lasting for over a year before PRP injection. She had no knee traumas in the last year. She reported recent smoking cessation, hypertension, and consumption of antithrombotic/antiplatelet drugs and vitamin D plus calcium supplements. She previously received intra-articular injections of hyaluronic acid for the same knee with no results. She received two intra-articular injections of PRGF-Endoret® one week apart, following the same procedure as reported for patient 1. Likewise patient 1, she was evaluated by using VAS, EQ-5D, Tegner Activity Scale, and KOOS-ADL.

All the pain-related scores did not change during the follow-up, with persisting mild-to-severe symptoms (Table 1). Concerning outcomes related to function and activity, Tegner showed a decrease, whereas KOOS remained stable. The utility value calculated from EQ-5D was 0.85 at both T0 and T1.

## Discussion

Injections of blood-derived products are a widely diffused treatment for KOA, and increasing evidence shows their safety and efficacy (Shen et al., 2017; Hohmann et al., 2020; Nie et al., 2021). However, while they can often improve symptoms, in many cases, pain, functional impairment, and reduced quality of life persist even after the injections. For these reasons, introducing this treatment into an overall rehabilitation approach whose efficacy has already been shown could improve the patient outcomes. In this perspective, blood-derived products would be a facilitator of patients’ recovery and find a better role in supporting more active treatments. While rehabilitation, mainly including therapeutic exercises, is considered an optimal ally for the conservative KOA treatment (Fransen et al., 2015), there is no current evidence that intra-articular injections of blood-derived products could enhance a specific exercise program's efficacy. It has not yet been defined which kind of exercises could be proposed safely after injections of blood-derived products. However, it is reasonable that an exercise program specifically tailored for these kinds of treatment could enhance their effect on pain and functional impairment in KOA patients. In our study, we propose a new treatment with the ambition to be adjuvant to PRGF injections and potentially even be synergic to it. We developed the program with two essential concepts in our mind: feasibility and safety. During the COVID-19 pandemic, hospitals should be visited as little as possible, and the home-based rehabilitation program is safer (DE Sire et al., 2021). Furthermore, many of our KOA patients (like patient 2 of our study) are young and active, and often do not have the time to go to the hospital. For these reasons, a 15- to 20-min home-based program performed twice a day seemed the right solution to maintain patients’ compliance while ensuring a valid number of repetitions. An article described the results of 4 patients undergoing StroMed and PRP injections and participating in a four-month rehabilitative program, including many outpatient sessions (Gibbs et al., 2015). After the program, patients’ pain and functional abilities improved; however, a 4-month outpatient rehabilitative program is not always feasible, especially in the current sanitary and socioeconomic situation, and different strategies should be considered. During the testing of our program, we realized that incorporating concepts of telemedicine and tele-rehabilitation could probably maintain the safety and feasibility of the program while possibly improving compliance and efficacy. Future studies should explore the potential of telemedicine, especially in young, active patients. The exercises prescribed were carefully chosen to be easy and feasible while maintaining efficacy. All the exercises should be executed while lying supine, eliminating the risk of falling. Isometric exercises do not need wide spaces to be performed, and they have been found beneficial in patients suffering from KOA (Anwer and Alghadir, 2014). While exercise #1 focuses mainly on strength, exercises #2 and #3 are designed to work also on proprioception and control of the affected limb, which is crucial for pain control in KOA (Jeong et al., 2019). Another important concept that led to the choice of these exercise was the possibility of a synergic effect with intra-articular injections. Actually, while PRGF injections could lead eventually to a partial restoration of damaged tissues, they cannot act directly on functional capabilities, an aspect of crucial important in osteoarthritis treatment. Improvement of tissues can theoretically enhance the improvement of functional capabilities due to the prescribed exercises. On the other hand, improvement of functional capabilities can potentially enhance tissue regeneration providing more physiological biomechanical stimuli (e.g., correct biomechanical load) (Bokaeian et al., 2021), thus creating a “virtuous circle.”

Due to the limited sample size, we can only evaluate the feasibility and safety of the program. However, the treatment of the two patients obtained the results we expected. The patients did not report any side effects due to the exercise program. The two cases studied were carefully selected in order to represent two different populations that can benefit from adding a short exercise program to PRP injections. While both patients suffered from KOA, patient 1 is an 85-year-old with severe KOA and reduced functional capabilities, as underlined by a baseline total KOOS score of 6.55, with a function sub-score of 5.88. On the other hand, patient 2 is a 59-year-old with better baseline functional performance with a total KOOS score of 60.12 and a function sub-score of 60.29. Indeed, the two patients represent two different populations that can potentially have different responses to therapy as well as different expectations. The difference in the results reflects the difference in baseline condition. Patient 1 dramatically improved at T2 in almost every outcome. It is interesting to notice that KOOS improved significantly. While exercises are often beneficial in KOA also for pain relief, especially in the long run, they are inherently linked to improving strength and proprioception needed to improve function and independence on ADL. Patient 2 showed less improvement. However, outcomes manifested a ceiling effect due to the relatively good clinical condition pretreatment. Furthermore, we prescribed the same set of exercises for both patients. However, the two patients had very different clinical conditions. It is possible that the intensity and degree of difficulty of exercises were enough to warrant a bettering in condition in a less performing patient such as patient 1, but were not enough for patient 2. Indeed, for future studies including young and active patients, functional tests with no ceiling effect, such as 6-minute walking test, 2-minute step-test, and 10-m walking test at maximum speed, should be implemented. Furthermore, exercise intensity should be tailored on functional capabilities of the individual patient.

Our study limitation is represented by the short follow-up. Unfortunately, the patients were treated right before the first pandemic lockdown in our country, and it was impossible to reach them out for longer time points. Certainly, future patients will have to be monitored at least until 12 months from the treatment.

Overall, the developed program seemed safe and was tolerated by the patients analyzed in the study, who performed it with good compliance. The results obtained motivated us to plan another study based on the same program, with the implementation of telemedicine and biomechanical evaluation to enhance compliance, efficacy, and outcomes that do not reach a ceiling effect in high-performance patients.

## Data Availability

The original contributions presented in the study are included in the article/Supplementary Material; further inquiries can be directed to the corresponding author.

## References

[B1] Ambrosio F. Rando T. A. (2018). The Regenerative Rehabilitation Collection: a Forum for an Emerging Field. Npj Regen. Med. 3, 1–2. 10.1038/s41536-018-0058-z 30374410 PMC6195606

[B2] Anwer S. Alghadir A. (2014). Effect of Isometric Quadriceps Exercise on Muscle Strength, Pain, and Function in Patients with Knee Osteoarthritis: a Randomized Controlled Study. J. Phys. Ther. Sci. 26, 745–748. 10.1589/jpts.26.745 24926143 PMC4047243

[B3] Block J. Miller L. (2013). An 8-week Multimodal Treatment Program Improves Symptoms of Knee Osteoarthritis: a Real-World Multicenter Experience. Por 4, 39. 10.2147/por.s53608 PMC5045015 27774023

[B4] Bokaeian H. R. Esfandiarpour F. Zahednejad S. Mohammadi H. K. Farahmand F. (2021). Effects of an Exercise Therapy Targeting Knee Kinetics on Pain, Function, and Gait Kinetics in Patients with Knee Osteoarthritis: A Randomized Clinical Trial. Adapt. Phys. Act. Q. APAQ 38, 377–395. 10.1123/apaq.2020-0144 33785660

[B5] Cole B. J. Seroyer S. T. Filardo G. Bajaj S. Fortier L. A. (2010). Platelet-rich Plasma: Where Are We Now and where Are We Going? Sports Health 2, 203–210. 10.1177/1941738110366385 23015939 PMC3445108

[B6] Collins N. J. Misra D. Felson D. T. Crossley K. M. Roos E. M. (2011). Measures of Knee Function: International Knee Documentation Committee (IKDC) Subjective Knee Evaluation Form, Knee Injury and Osteoarthritis Outcome Score (KOOS), Knee Injury and Osteoarthritis Outcome Score Physical Function Short Form (KOOS-PS), Knee Ou. Arthritis Care Res. 63, S208–S228. 10.1002/acr.20632 PMC4336550 22588746

[B7] DE Sire A. Andrenelli E. Negrini F. Patrini M. Lazzarini S. G. Ceravolo M. G. (2021). Rehabilitation and COVID-19: a Rapid Living Systematic Review by Cochrane Rehabilitation Field Updated as of December 31st, 2020 and Synthesis of the Scientific Literature of 2020. Eur. J. Phys. Rehabil. Med. 57, 181-188. 10.23736/S1973-9087.21.06870-2 33599442

[B8] DeLong J. M. Russell R. P. Mazzocca A. D. (2012). Platelet-rich Plasma: the PAW Classification System. Arthrosc. J. Arthroscopic Relat. Surg. 28, 998–1009. 10.1016/j.arthro.2012.04.148 22738751

[B9] Deshpande B. R. Katz J. N. Solomon D. H. Yelin E. H. Hunter D. J. Messier S. P. (2016). Number of Persons with Symptomatic Knee Osteoarthritis in the US: Impact of Race and Ethnicity, Age, Sex, and Obesity. Arthritis Care Res. 68, 1743–1750. 10.1002/acr.22897 PMC5319385 27014966

[B10] Dragoo J. L. Braun H. J. Durham J. L. Ridley B. A. Odegaard J. I. Luong R. (2012). Comparison of the Acute Inflammatory Response of Two Commercial Platelet-Rich Plasma Systems in Healthy Rabbit Tendons. Am. J. Sports Med. 40, 1274–1281. 10.1177/0363546512442334 22495144

[B11] Flaumenhaft R. Koseoglu S. (2017). Platelet Contents in Molecular and Cellular Biology of Platelet Formation: Implications in Health and Disease. (Springer International Publishing), 133–152. 10.1007/978-3-319-39562-3_6

[B12] Fransen M. Mcconnell S. Harmer A. R. Van der Esch M. Simic M. Bennell K. L. (2015). Exercise for Osteoarthritis of the Knee. Cochrane Database Syst. Rev. 1, CD004376. 10.1002/14651858.CD004376.pub3 25569281 PMC10094004

[B13] Gagnier J. J. Kienle G. Altman D. G. Moher D. Sox H. Riley D. (2013). The CARE Guidelines: Consensus-Based Clinical Case Reporting Guideline Development. Glob. Adv. Health Med. 2, 38–43. 10.7453/gahmj.2013.008 PMC3833570 24416692

[B14] Gibbs N. Diamond R. Sekyere E. Thomas W. (2015). Management of Knee Osteoarthritis by Combined Stromal Vascular Fraction Cell Therapy, Platelet-Rich Plasma, and Musculoskeletal Exercises: a Case Series. Jpr 8, 799–806. 10.2147/JPR.S92090 PMC4644167 26609244

[B15] Haq I. Murphy E. Dacre J. (2003). Osteoarthritis. Postgrad. Med. J. 79, 377–383. 10.1136/pmj.79.933.377 12897215 PMC1742743

[B16] Hohmann E. Tetsworth K. Glatt V. (2020). Is Platelet-Rich Plasma Effective for the Treatment of Knee Osteoarthritis? A Systematic Review and Meta-Analysis of Level 1 and 2 Randomized Controlled Trials. Eur. J. Orthop. Surg. Traumatol. 30, 955–967. 10.1007/s00590-020-02623-4 32060630

[B17] Hunter D. J. Neogi T. Hochberg M. C. (2011). Quality of Osteoarthritis Management and the Need for Reform in the US. Arthritis Care Res. 63, 31–38. 10.1002/acr.20278 20583113

[B18] Hurley M. Dickson K. Hallett R. Grant R. Hauari H. Walsh N. (2018). Exercise Interventions and Patient Beliefs for People with Hip, Knee or Hip and Knee Osteoarthritis: A Mixed Methods Review. Cochrane Database Syst. Rev. 4 (4), CD010842. 10.1002/14651858.CD010842.pub2 29664187 PMC6494515

[B19] Jeong H. S. Lee S.-C. Jee H. Song J. B. Chang H. S. Lee S. Y. (2019). Proprioceptive Training and Outcomes of Patients with Knee Osteoarthritis: A Meta-Analysis of Randomized Controlled Trials. J. Athl. Train. 54, 418–428. 10.4085/1062-6050-329-17 30995119 PMC6522092

[B20] Kennedy J. A. Mohammad H. R. Mellon S. J. Dodd C. A. F. Murray D. W. (2020). Age Stratified, Matched Comparison of Unicompartmental and Total Knee Replacement. The Knee 27, 1332–1342. 10.1016/j.knee.2020.06.004 33010745

[B21] Kolasinski S. L. Neogi T. Hochberg M. C. Oatis C. Guyatt G. Block J. (2020). 2019 American College of Rheumatology/Arthritis Foundation Guideline for the Management of Osteoarthritis of the Hand, Hip, and Knee. Arthritis Care Res. 72, 149–162. 10.1002/acr.24131 PMC11488261 31908149

[B22] Lana J. F. Huber S. C. Purita J. Tambeli C. H. Santos G. S. Paulus C. (2019). Leukocyte-rich PRP versus Leukocyte-Poor PRP - the Role of Monocyte/macrophage Function in the Healing cascade. J. Clin. Orthopaedics Trauma 10, S7–S12. 10.1016/j.jcot.2019.05.008 PMC6823808 31700202

[B23] Le A. D. K. Enweze L. DeBaun M. R. Dragoo J. L. (2018). Current Clinical Recommendations for Use of Platelet-Rich Plasma. Curr. Rev. Musculoskelet. Med. 11, 624–634. 10.1007/s12178-018-9527-7 30353479 PMC6220007

[B24] Man G. S. Mologhianu G. (2014). Osteoarthritis Pathogenesis - a Complex Process that Involves the Entire Joint. J. Med. Life 7, 37–41. PMC3956093 24653755

[B25] Nguyen R. T. Borg-Stein J. McInnis K. (2011). Applications of Platelet-Rich Plasma in Musculoskeletal and Sports Medicine: An Evidence-Based Approach. PM&R 3, 226–250. 10.1016/j.pmrj.2010.11.007 21402369

[B26] Nie L.-Y. Zhao K. Ruan J. Xue J. (2021). Effectiveness of Platelet-Rich Plasma in the Treatment of Knee Osteoarthritis: A Meta-Analysis of Randomized Controlled Clinical Trials. Orthopaedic J. Sports Med. 9, 232596712097328. 10.1177/2325967120973284 PMC7930657 33718505

[B27] Roos E. M. Roos H. P. Lohmander L. S. Ekdahl C. Beynnon B. D. (1998). Knee Injury and Osteoarthritis Outcome Score (KOOS)-Development of a Self-Administered Outcome Measure. J. Orthop. Sports Phys. Ther. 28, 88–96. 10.2519/jospt.1998.28.2.88 9699158

[B28] Sánchez M. Fiz N. Azofra J. Usabiaga J. Aduriz Recalde E. Garcia Gutierrez A. (2012). A Randomized Clinical Trial Evaluating Plasma Rich in Growth Factors (PRGF-Endoret) versus Hyaluronic Acid in the Short-Term Treatment of Symptomatic Knee Osteoarthritis. Arthrosc. J. Arthroscopic Relat. Surg. 28, 1070–1078. 10.1016/j.arthro.2012.05.011 22840987

[B29] Scalone L. Cortesi P. A. Ciampichini R. Belisari A. D’Angiolella L. S. Cesana G. (2013). Italian Population-Based Values of EQ-5D Health States. Value in Health 16, 814–822. 10.1016/j.jval.2013.04.008 23947975

[B30] Shen L. Yuan T. Chen S. Xie X. Zhang C. (2017). The Temporal Effect of Platelet-Rich Plasma on Pain and Physical Function in the Treatment of Knee Osteoarthritis: Systematic Review and Meta-Analysis of Randomized Controlled Trials. J. Orthop. Surg. Res. 12. 10.1186/s13018-017-0521-3 PMC5260061 28115016

[B31] Spitaels D. Mamouris P. Vaes B. Smeets M. Luyten F. Hermens R. (2020). Epidemiology of Knee Osteoarthritis in General Practice: a Registry-Based Study. BMJ Open 10, e031734. 10.1136/bmjopen-2019-031734 PMC7044813 31964664

[B32] Tegner Y. Lysholm J. (1985). Rating Systems in the Evaluation of Knee Ligament Injuries. Clin. Orthop. Relat. Res., 43–49. 10.1097/00003086-198509000-00007 4028566

[B33] van Reenen M. Janssen B. Oppe M. Kreimeier S. Greiner W. Stolk E. (2020). EuroQol Research Foundation. EQ-5d-Y User Guide. Available from: https://euroqol.org/publications/user-guides.

[B34] Vos T. Flaxman A. D. Naghavi M. Lozano R. Michaud C. Ezzati M. (2012). Years Lived with Disability (YLDs) for 1160 Sequelae of 289 Diseases and Injuries 1990-2010: A Systematic Analysis for the Global Burden of Disease Study 2010. Lancet 380, 2163–2196. 10.1016/S0140-6736(12)61729-2 23245607 PMC6350784

[B35] Wewers M. E. Lowe N. K. (1990). A Critical Review of Visual Analogue Scales in the Measurement of Clinical Phenomena. Res. Nurs. Health 13, 227–236. 10.1002/nur.4770130405 2197679

[B36] Woodell-May J. Steckbeck K. King W. (2021). Potential Mechanism of Action of Current Point-of-Care Autologous Therapy Treatments for Osteoarthritis of the Knee-A Narrative Review. Ijms 22, 2726. 10.3390/ijms22052726 33800401 PMC7962845

[B37] Xing D. Wang B. Liu Q. Ke Y. Xu Y. Li Z. (2016). Intra-articular Hyaluronic Acid in Treating Knee Osteoarthritis: A PRISMA-Compliant Systematic Review of Overlapping Meta-Analysis. Sci. Rep. 6. 10.1038/srep32790 PMC5018721 27616273

[B38] Yan R. Gu Y. Ran J. Hu Y. Zheng Z. Zeng M. (2017). Intratendon Delivery of Leukocyte-Poor Platelet-Rich Plasma Improves Healing Compared with Leukocyte-Rich Platelet-Rich Plasma in a Rabbit Achilles Tendinopathy Model. Am. J. Sports Med. 45, 1909–1920. 10.1177/0363546517694357 28301205

